# Mathematical simulations of paediatric haemodynamics in isolated ventricular septal defect

**DOI:** 10.1098/rsif.2026.0187

**Published:** 2026-10-14

**Authors:** Mitchel J. Colebank, Alfonso Limon, Anthony Chang, Brandon Wong, Wyman Lai, Hamilton Baker

**Affiliations:** ^1^ Department of Mathematics, University of South Carolina, Columbia, SC, USA; ^2^ Department of Biomedical Engineering, University of South Carolina, Columbia, SC, USA; ^3^ Rady Children's Health of Orange County, Orange, CA, USA; ^4^ University of California Irvine, Irvine, CA, USA; ^5^ Medical University of South Carolina, Charleston, SC, USA

**Keywords:** mathematical modelling, congenital heart disease, cardiovascular simulation, ventricular septal defect

## Abstract

Computer modelling of the cardiovascular system has the potential to revolutionize personalized medical care. This is especially promising for congenital heart defects, such as ventricular septal defect (VSD), a hole between the two ventricles of the heart. However, relatively few studies have built computer models for VSD, nor have they considered how natural adaptation to the cardiovascular system with age might interact with the presence of a small-, medium- or large-sized VSD. Here, we combine a lumped-parameter model of the cardiovascular system with two key modelling components: a size-dependent resistance dictating shunt flow between the two ventricles and age-dependent scaling relationships for the systemic and pulmonary circulations. Our results provide insight into changes in haemodynamic conditions with various VSD sizes. We investigate the combined effects of VSD size, vascular parameters and age, showing distinct differences with these three factors. This study lays the necessary foundation for studying VSD and towards building digital shadows and digital twins for managing VSD in paediatrics.

## 1. Introduction

Mathematical models are an increasingly recognized future tool for the management of cardiovascular disease. The most notable example includes the use of computational fluid dynamics models to predict coronary artery haemodynamics and assist in subject-specific treatment of ischaemic conditions [1]. These computational frameworks have potential in managing complex congenital heart diseases [2–5]. While fluid dynamics models have high spatial fidelity, they often come at elevated computation time which may be infeasible for clinical decision-making. Thus, lumped-parameter models, which describe flow through compartments and interactions between cardiac and vascular segments, are attractive alternatives. These models have also received attention in predicting neonatal and paediatric haemodynamics under the effects of congenital heart defects and surgical correction [6–10].

The most common congenital heart defect is ventricular septal defect (VSD), which affects approximately 0.3% of all live births and accounts for approximately one-third of all congenital heart disease diagnoses [11–13]. The defect, a hole in the interventricular septum, which separates the left ventricle (LV) and right ventricle (RV), can drastically change cardiac and vascular haemodynamics depending on the defect size and location. Small, high-resistance VSDs (typically less than 3 mm in diameter at birth) often close spontaneously with increasing age of the subject, whereas subjects with large VSDs at birth (greater than 6 mm in diameter) undergo surgical intervention [11], as they can cause congestive heart failure, impaired growth and eventual pulmonary vascular disease if left untreated. Medium-sized VSDs (3–6 mm in diameter at birth) may or may not close naturally depending on the haemodynamic status of the subject. The latter can cause growth and remodelling and cardiovascular complications (e.g. pulmonary hypertension [11,14,15]). Gold standard diagnosis and management of VSD uses echocardiography measurements to assess VSD size, pulmonary-to-systemic flow (*Q_p_*/*Q_s_*) ratio, left atrial and LV size and pulmonary haemodynamic metrics to assess appropriate standard of care [11,14–16]. However, VSD diagnosis is associated with significant increases in mortality owing to heart conditions at late age points [16]. To date, relatively few computational studies have simulated haemodynamics in the setting of a VSD [9,10], nor have studies considered how natural adaptation with growth in combination with a VSD alters haemodynamics.

To address this knowledge gap, this study uses a lumped-parameter model of the cardiovascular system with age-based parameter adaptation rules to simulate the effects of VSD. We combine an eight-compartment model with previously established allometric- and maturation-based parameter scaling of the systemic and pulmonary vasculature [8]. We simulate haemodynamics from one day to two years of age in a normal physiological paediatric case, as well as in small, medium and large VSDs. We consider the effects of elevated pulmonary vascular resistance (PVR), consistent with the development of pulmonary hypertension, as well as uncertainty in maturation-based adaptation and total blood volume. Our results provide an initial step towards simulating age-dependent haemodynamic function with isolated VSD, which is needed as we move towards using predictive modelling in managing congenital heart diseases.

## 2. Material and methods

### 2.1. Mathematical model of the cardiovascular system and ventricular septal defect

This study uses systems of ordinary differential equations (ODEs) that simulate dynamic pressure *p*(*t*) (mmHg), volume *V*(*t*) (ml) and flow *q*(*t*) (ml s^−1^) using reformulations of electrical circuit equations [17,18]. The model includes four heart chambers: the left atrium (LA), LV, right atrium (RA) and RV. We also include systemic arterial (SA), systemic venous (SV), pulmonary arterial (PA) and pulmonary venous (PV) compartments for the circulation. The two atrioventricular (tricuspid and mitral) and two semilunar (pulmonary and aortic) valves are modelled as diodes, closing when the pressure gradient across the valve is negative. A schematic of the model is provided in figure 1. Though this neglects inertial effects of valve dynamics, it can recapitulate valvular-flow profiles in the absence of regurgitation. The differential equation for volume and algebraic relationships for compartment and valve flow, compartment pressure and heart pressure are given by

(2.1)dVidt=qin−qout,(2.2)qc,i=ΔpiRi,qvali=max(0,ΔpiRi)(2.3)pc,i=Vc,i(t)Ci,(2.4)ph,i(t)=Eh,i(t)Vh,i(t)

respectively. Equation (2.1) describes changes in stressed volume *V_i_*(*t*) (the blood volume that generates transmural pressure) in compartment *i*, which is dictated by flow conservation using equation (2.2). Non-valve flow dynamics are proportional to the ratio of the pressure gradient Δ*p_i_* and the resistance, *R_i_* (mmHg s ml^−1^), between compartments [18]. Valve flow equations are similar but set to zero when pressure gradients become negative [17]. Pressure in the vascular compartments, *p*_*c*,*i*_, are described by equation (2.3) and are proportional to the ratio of vascular compartment volume (*V*_*c*,*i*_) and vascular compliance, *C_i_* (ml mmHg^−1^).

**Figure 1.**
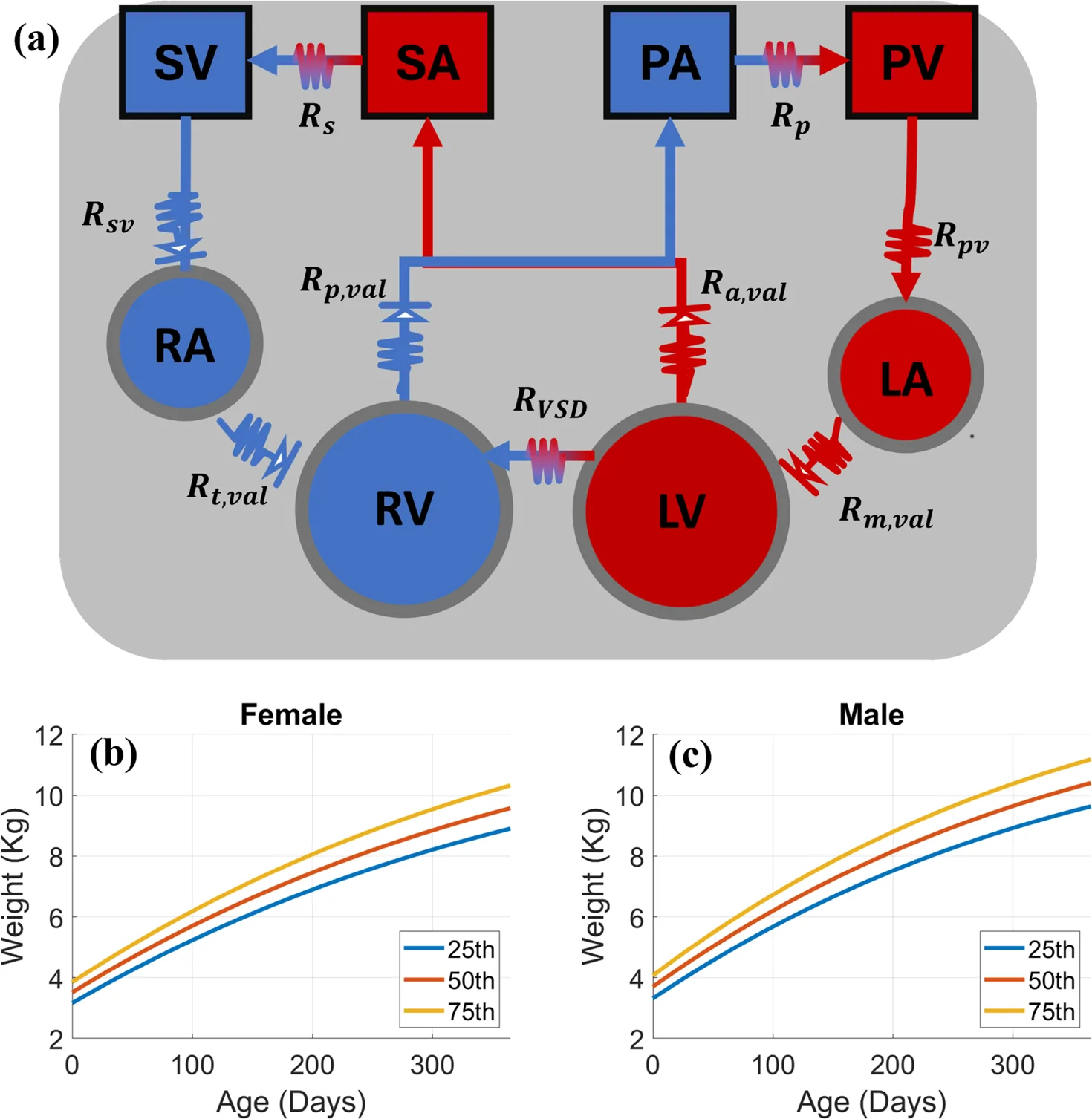
(a) Schematic of the mathematical model. The left atrium (LA), left ventricle (LV), right atrium (RA) and right ventricle (RV) are described by elastance functions and connected to the systemic and pulmonary arteries (SA and PA) and veins (SV and PV) through resistors. The circulation is modelled as a compliance chamber. (b and c) Growth trajectories for female and male individuals (respectively) through 1 year of life based on Centers for Disease Control (CDC) data.

Pressures in each heart chamber, *p*_*h*,*i*_, are the product of cardiac elastance, *E*_*h*,*i*_ (mmHg ml^−1^), and the blood volume in the heart, *V*_*h*,*i*_, as shown in equation (2.4). The elastance function driving heart contraction is described by a piecewise cosine function [17],

(2.5) $$E_{h,i}\left(t\right)=\{E_{min,i}+\;\begin{array}{ccc} & E_{min,i}+\;\frac{E_{max,i}-E_{min,i}}{2}\left(1-\cos\left(\frac{\tilde{t}}{T_{max,i}}\right)\right),\;\tilde{t}\in \left[0,T_{max,i}\right] & \\ & \frac{E_{max,i}-E_{min,i}}{2}\left(1+\cos\left(\frac{\tilde{t}-T_{max,i}}{T_{min,i}-T_{max,i}}\right)\right),\;\;\tilde{t}\in \left(T_{max,i},T_{min,i}\right] & \\ & \; & \\ & \;E_{min,i},\;\text{else} & \end{array}\;$$

where $\tilde{t}=mod(t,T)$ is the cardiac cycle time modulated by the cardiac cycle length, *T*  =  60/HR (s), where HR is the heart rate in beats per minute. The elastance function oscillates between the minimum elastance, *E*_min,*i*_, and maximum elastance, *E*_max,*i*_, for each heart compartment *i*  =  LA, LV, RA, RV. The parameters *T*_max,*i*_ and *T*_min,*i*_ (s) correspond to when the cardiac elastance functions reach their maximum and minimum values, corresponding to contraction during systole and the relaxation back to diastole [17]. An additional offset parameter, *T*_off_ (s), is prescribed to the atria so that atrial contraction precedes ventricular contraction [17].

To simulate flow through a VSD, we include an additional resistor between the LV and RV [19]. The flow across the ventricular defect, *q*_VSD_, is modelled by reduced-order orifice flow,

(2.6) $$\;q_{VSD}=\;\text{sign}\left(\Delta p\right)\cdot \pi r_{VSD}^{2}\;\sqrt{p^{*}\cdot \left(2\left|\Delta p\right|/\rho \right)},\;\Delta p\;=p_{LV}\;\left(t\right)-p_{RV}\left(t\right),$$

where *r*_VSD_ is the VSD radius (cm), ρ  =  1.055 (g cm^−3^) is the blood density, and p* =  1333.22 (g cm^−1^ s^2^) is a conversion factor between mmHg and centimetre–grams–seconds (CGS) units. The sign convention implies that flow from the LV to the RV is considered positive, while flow from the RV to the LV is considered negative. The volume equations for the LV and RV are modified to

(2.7) $$\frac{dV_{LV}}{dt}=q_{M,Val}\;-q_{A,Val}-q_{VSD}$$

and

(2.8) $$\frac{dV_{RV}}{dt}=q_{T,Val}\;-q_{P,Val}+q_{VSD},$$

respectively, where *q*_*M*,Val_, *q*_*A*,Val_, *q*_*T*,Val_ and *q*_*P*,Val_ are the mitral, aortic, tricuspid and pulmonic valve flows (ml s^−1^), respectively. We parametrize the VSD using a orifice-flow coefficient element as $K_{VSD}=(\pi r_{VSD}^{2}\cdot \sqrt{p^{*}\cdot 2/\rho }{)}^{-1}\;$ by reformulating equation (2.6) and use this resistance in our analysis. All model parameters and outputs are computed in units of mmHg, ml and seconds.

### 2.2. Parametrization of the model

The system of ODEs is driven by resistance, compliance, elastance and timing parameters. We begin by parametrizing the model for a reference adult of either sex. We then scale the adult parameters to neonate and paediatric values, as discussed in §2.3. The parameters and their *a priori* calculations can be found in table 1.

**Table 1.** Nominal values and parameters for adult females/males used in this study.

parameter	description	units	nominal value or equation
*nominal volumes* (adult female/male)			
$V_{LA}^{0}$	initial LA volume	ml	20/25
$V_{LV}^{0}$	initial LV volume	ml	100/120
$V_{SA}^{0}$	initial SA volume	ml	182.25/202.5
$V_{SV}^{0}$	initial SV volume	ml	675/750
$V_{RA}^{0}$	initial RA volume	ml	15/25
$V_{RV}^{0}$	initial RV volume	ml	100/120
$V_{PA}^{0}$	initial PA volume	ml	234.9/261
$V_{PV}^{0}$	initial PV volume	ml	49.5/55
*nominal pressures* (adult female/male)			
*p*_SA,max_	maximum SA pressure	mmHg	110/120
*p*_SA,min_	minimum SA pressure	mmHg	70/80
${\bar{p}}_{SA}$	mean SA pressure	mmHg	83.33/93.33
*p*_PA,max_	maximum PA pressure	mmHg	17/20
*p*_PA,min_	minimum PA pressure	mmHg	5/8
${\bar{p}}_{PA}$	mean PA pressure	mmHg	12/13
${\bar{p}}_{SV}$	mean SV pressure	mmHg	8/10
${\bar{p}}_{PV}$	mean PV pressure	mmHg	4/4
*p*_LA,max_	maximum LA pressure	mmHg	13/16
*p*_LA,min_	minimum LA pressure	mmHg	3/3
*p*_LV,max_	maximum LV pressure	mmHg	111/121
*p*_LV,min_	minimum LV pressure	mmHg	5/5
*p*_RA,max_	maximum RA pressure	mmHg	6/8
*p*_RA,min_	minimum RA pressure	mmHg	2/2
*p*_RV,max_	maximum RV pressure	mmHg	18/21
*p*_RV,min_	minimum RV pressure	mmHg	3/3
*vascular system parameters*			
*R*_*a*,val_	aortic valve resistance	mmHg s ml^−1^	0.1/CO
*R*_*m*,val_	mitral valve resistance	mmHg s ml^−1^	0.5/CO
*R*_*p*,val_	pulmonic valve resistance	mmHg s ml^−1^	0.1/CO
*R*_*t*,val_	tricuspid valve resistance	mmHg s ml^−1^	0.5/CO
*R*_vc_	vena cava resistance	mmHg s ml^−1^	$({\bar{p}}_{SV}-p_{RA,min})/CO$
*R*_pv_	pulmonary venous resistance	mmHg s ml^−1^	$({\bar{p}}_{PV}-p_{LA,min})/CO$
*R_s_*	systemic circulation resistance	mmHg s ml^−1^	$({\bar{p}}_{SA}-p_{SV},)/CO$
*R_p_*	pulmonary circulation resistance	mmHg s ml^−1^	$({\bar{p}}_{PA}-{\bar{p}}_{PV})/CO$
*K*_VSD_	ventricular septal defect orifice-flow coefficient	mmHg^-1/2^ s ml^−1^	$(\pi r_{VSD}^{2}\cdot \sqrt{p^{*}\cdot 2/\rho }{)}^{-1}$
*C*_sa_	compliance of systemic arteries	ml mmHg^−1^	Stroke Volume/(*p*_SA,max_ − *p*_SA,min_)
*C*_sv_	compliance of systemic veins	ml mmHg^−1^	$V_{sv}/{\bar{p}}_{sv}$
*C*_pa_	compliance of pulmonary arteries	ml mmHg^−1^	Stroke Volume/(*p*_PA,max_ − *p*_PA,min_)
*C*_pv_	compliance of pulmonary veins	ml mmHg^−1^	$V_{pv}/{\bar{p}}_{pv}$
*cardiac parameters*			
*E*_max,LA_	maximum LA elastance	mmHg ml^−1^	*p*_LA,max_/*V*_LA,min_
*E*_min,LA_	minimum LA elastance	mmHg ml^−1^	(*p*_LA,min_ + *p*_LA,max_)/(*V*_LA,min_ + *V*_LA,max_)
*E*_max,LV_	maximum LV elastance	mmHg ml^−1^	*p*_LV,max_/*V*_LV,min_
*E*_min,LV_	minimum LV elastance	mmHg ml^−1^	*p*_LV,min_/*V*_LV,max_
*E*_max,RA_	maximum RA elastance	mmHg ml^−1^	*p*_RA,max_/*V*_RA,min_
*E*_min,RA_	minimum RA elastance	mmHg ml^−1^	(*p*_RA,min_ + *p*_RA,max_)/(*V*_RA,min_ + *V*_RA,max_)
*E*_max,RV_	maximum RV elastance	mmHg ml^−1^	*p*_RV,max_/*V*_RV,min_
*E*_min,RV_	minimum RV elastance	mmHg ml^−1^	*p*_RV,min_/*V*_RV,max_
*T*_max,LA_	time of maximum LA elastance	s	0.1 · *T*
*T*_min,LA_	time of minimum LA elastance	s	0.2 · *T*
*T*_max,LV_	time of maximum LV elastance	s	0.25 · *T*
*T*_min,LV_	time of minimum LV elastance	s	0.55 · *T*
*T*_max,RA_	time of maximum RA elastance	s	0.1 · *T*
*T*_min,RA_	time of minimum RA elastance	s	0.2 · *T*
*T*_max,RV_	time of maximum RV elastance	s	0.25 · *T*
*T*_min,RV_	time of minimum RV elastance	s	0.55 · *T*
*T*_off_	offset time for atrial contraction	s	0.15 · *T*

CO: cardiac output; LA: left atrium; LV: left ventricle; PA: pulmonary arteries; PV: pulmonary veins; RA: right atrium; RV: right ventricle; SA: systemic arteries; SV: systemic veins.

We assume that the adult blood volume is 4500 ml in females and 5000 ml in males [20]. Each model compartment is given a percentage of the total blood volume. A fraction of this is considered the stressed, circulating volume, similar to previous studies [17]. A list of total and stressed volume percentages can be found in table 1. We assume that the pressures are similar between female/male, with slightly lower systemic and pulmonary pressures in females consistent with the literature [20]. We also assume a stroke volume of 70 ml in adult females versus 80 ml in adult males and heart rates of 80 and 70 beats per minute for females and males, respectively. This provides a cardiac output (CO) of 5.6 l min^−1^ (or 93.33 ml s^−1^) for females and males. We then use reference values for compartment pressures and literature-derived distributions for the total blood volume [17,20] to construct initial parameter estimates for the model.

Resistance values, *R_i_*, are set as the pressure gradient across the compartment divided by the cardiac output. Valvular resistances are fixed at smaller values to ensure proper filling during diastole and avoid pressure differentials seen in valvular stenoses [17]. The resistance of the VSD is set to 1 × 10^10^ (mmHg ml^−1^) in the absence of a defect or defined as shown in §2.1. Systemic and PA compliances, *C*_sa_ and *C*_pa_, are defined as stroke volume over pulse pressure (i.e. the difference between systolic and diastolic pressures). Systemic and PV compliances, *C*_sv_ and *C*_pv_, are defined as compartment volume divided by the mean pressure in the respective compartments. The ventricular end-diastolic elastance, *E*_min_, and end-systolic elastance, *E*_max_, are defined as *E*_min_ = *p*_dias_ /*V*_dias_ and *E*_max_ = *p*_sys_ /*V*_sys_, respectively. For atria, we use the same relationship for *E*_max_ but instead use *E*_min_ = (*p*_sys_ + *p*_dias_ )/(*V*_sys_ + *V*_dias_)  for minimum atrial elastance to provide larger pressure developments during atrial filling [17]. We assume that peak contraction, *T*_max_, and the start of cardiac diastole, *T*_min_, occur 25% into the cardiac cycle and 55% into the cardiac cycle, that is *T*_max_ =  0.25 · *T* and *T*_min_ =  0.55 · *T*, respectively. We assume that atrial contraction occurs 0.15 · *T* before the start of ventricular contraction and then make *T*_max_ =  0.10 · *T* and *T*_min_ =  0.20 · *T* in the atria. Table 1 provides a summary of the model parameters, their units, interpretations and calculation methods.

### 2.3. Age-dependent parametrization

We modify the framework presented by Hiebing *et al.* [8] for generating age-specific parametrization using allometric and nonlinear scaling of the adult parameters. To begin, we fit a cubic function to paediatric age–weight data from the Centers for Disease Control online database to provide the 25th, 50th and 75th percentile weight for any age group within the first two years of life. We include both male and female growth trajectories, though the general shapes of the growth curves (shown in figure 1) are nearly identical, with females having lower weight magnitudes. Allometric scaling is driven by these weights using

(2.9) $$\;X_{i}=X_{0}\;\cdot {\left(\frac{M_{i}}{M_{0}}\right)}^{b},$$

where *X_i_* is the scaled parameter, *X*_0_ is the reference value and *M_i_* and *M*_0_ are the current and initial body weights (kg) of the patient. The exponent *b* varies between different parameters of the model, as described in table 2. We assume that *X*_0_ and *M*_0_ correspond to the adult parametrization, as shown in table 1. We employ this allometric scaling relationship for the total blood volumes and all model parameters except pulmonary and SA resistance and compliance.

**Table 2.** List of adaptation parameters for the growth rules and parameter adaptations.

parameter	description	units	value (ref)
*allometric scaling*			
*b*_BV_	blood volume	ND	1.0 [8]
*b_HR_*	heart rate	ND	− 0.25 [8]
$b_{E_{max}}$	maximum elastance (*E*_max_)	ND	− 1.0 [8]
$b_{E_{min}}$	minimum elastance (*E*_min_)	ND	− 0.5 (determined)
$b_{T_{max}}$	maximum ventricular elastance timing (*T*_max_)	ND	− 0.07 [8]
*b_R_*	vascular resistance (*R_p_*,*R*_vc_,*R*_pv_)	ND	− 0.75 [8]
$b_{R_{s}}$	systemic vascular resistance (*R_s_*)	ND	− 0.5 (determined)
$b_{C_{v}}$	venous compliance (*C_sv_* and *C_pv_*)	ND	1.0 [8]
$b_{C_{A}}$	arterial compliance (*C*_sa_ and *C*_pa_)	ND	− 1.33 [8]
*maturation parameters* (systemic and pulmonary resistance)			
*g*_*s*,1_	early-age systemic vascular resistance	mmHg s ml^−1^	3.8 (determined)
*g*_*s*,2_	systemic vascular resistance time constant	days	50 (determined)
*g*_*s*,3_	systemic vascular resistance scaling term	ND	1.1 (determined)
*g*_*p*,1_	early-age pulmonary vascular resistance	mmHg s ml^−1^	4.0 (determined)
*g*_*p*,2_	pulmonary vascular resistance time constant	days	1.0 (determined)
*g*_*p*,3_	pulmonary vascular resistance scaling term	ND	2.0 (determined)

The maturation approach presented by Hiebing *et al.* [8] simulates the nonlinear changes in pulmonary and SA parameters during the initial transition of the neonatal and paediatric circulation. The scaled PVR, *R_p_*, and systemic vascular resistance (SVR), *R_s_*, are described as a function of time *t* in days,

(2.10)Rp(t)=gp,11+(t/gp,2)gp,3+Rp(0)⋅(MiM0)bRp⋅((t/gp,2)gp,31+(t/gp,2)gp,3)(2.11)Rs(t)=gs,11+(t/gs,2)gs,3+Rs(0)⋅(MiM0)bRs⋅((t/gs,2)gs,31+(t/gs,2)gs,3).

We use a different mathematical model to Hiebing *et al.*; hence, we modify the *g* parameters from equations (2.10) and (2.11) to fit our approach. This provides *g*_*p*,1_ =  4.0 (mmHg s ml^−1^), *g*_*p*,2_ =  1.0 (days) and *g*_*p*,3_ =  2.0 for the pulmonary circulation and *g*_*s*,1_ =  3.8 (mmHg s ml^−1^), *g*_*s*,2_ =  50 (days) and *g*_*s*,3_ =  1.1 for the systemic circulation. We also set *b*_pa_ = −0.75 like the other resistance parameters but use *b_sys_* = −0.5 for a more gradual change in SVR over time. These values provide haemodynamic predictions from the model that are consistent with the literature over two years of life (shown later) [21–29]. We use equations (2.9)–(2.11) to provide age-dependent parameters as a function of age for model simulations across two years of life.

### 2.4. Simulating ventricular septal defect with pulmonary vascular dysfunction

One possible complication of VSD is elevated *R_p_* and reduced *C*_pa_ with ageing. This is attributed in part to elevated *Q_p_*/*Q_s_* ratios, where large pulmonary flow increases shear stress magnitude on the pulmonary arteries and causes out-of-proportion increases in resistance owing to endothelial dysfunction [30,31]. We investigate this in two scenarios. In the first case, we assume pulmonary vascular parameters adapt using equation (2.10) during the first 30 days of life, but that *R_p_* and *C*_pa_ then remain at the fixed value, *R_p_*(30) and *C*_pa_(30), while other parameters adapt with age. In the second case, we assume that pulmonary adaptation only occurs up until 3 days of age and fix *R_p_* and *C*_pa_ at *R_p_*(3) and *C*_pa_(3), respectively. We investigate how these changes affect haemodynamics at time points up to 2 years of age.

### 2.5. Quantifying uncertainty in vascular adaptation

The maturation functions in equations (2.10) and (2.11) dictate how parameters adapt with age but are based on observations across multiple datasets. Thus, we investigate the predictive uncertainty in our model by sampling the maturation parameters, as well as the total blood volumes, and simulating growth trajectories. We simulate 100 different trajectories from birth to 2 years of age by sampling each *g* parameter from equations (2.10) and (2.11) from a uniform distribution with bounds ± 30% of the nominal value, that is

(2.12) $$g_{i.j}^{k}∼U\left(\frac{2g_{i,j}}{3},\frac{4g_{i,j}}{3}\right),\;i\;=\;p,s,\;j\;=\;1,2,3,$$

where *g*_*i*,*j*_ is the nominal value of the maturation parameters for the pulmonary or systemic system. We apply the same uncertainty bounds to the total blood volume to simulate differences in subject size. We examine how time-dependent cardiac function (i.e. pressure and volume curves) and scalar metrics (mean pulmonary artery pressure (mPAP), mean systemic arterial pressure (MAP) and the ratio of cycle-averaged pulmonic and aortic flow (*Q_p_*/*Q_s_*)) vary when adaptation curves are treated as uncertain across 2 years of age. We encode and simulate the mathematical model in MATLAB (MathWorks and Natick, MA) v. 2024a using the *ode15s* solver with relative and absolute tolerances of 1 × 10^−6^, using 30 heartbeats to achieve convergence.

## 3. Results

### 3.1. Comparison of the paediatric model to data and adult simulations

Figure 2 shows the predicted MAP and mPAP from the mathematical model over 2 years of life in both sexes with adaptation according to equations (2.9)–(2.11). We see that MAP increases with age, while mPAP has a drastic drop during the first several days of development consistent with reduced PVR. These predictions are compared with literature data [21–29] as originally presented by Hiebing *et al.* [8]. Overall, model predictions are within the variability reported from previous studies. We also show end-systolic pressure (ESP), end-systolic volume (ESV), end-diastolic pressure (EDP) and end-diastolic volume (EDV) in both ventricles in figure 2c–f. Both heart chambers show an increase in ESV and EDV, with minor sex differences. Predictions of LV ESV and RV ESV are smaller than data reported for subjects closer to 2 years of age.

**Figure 2.**
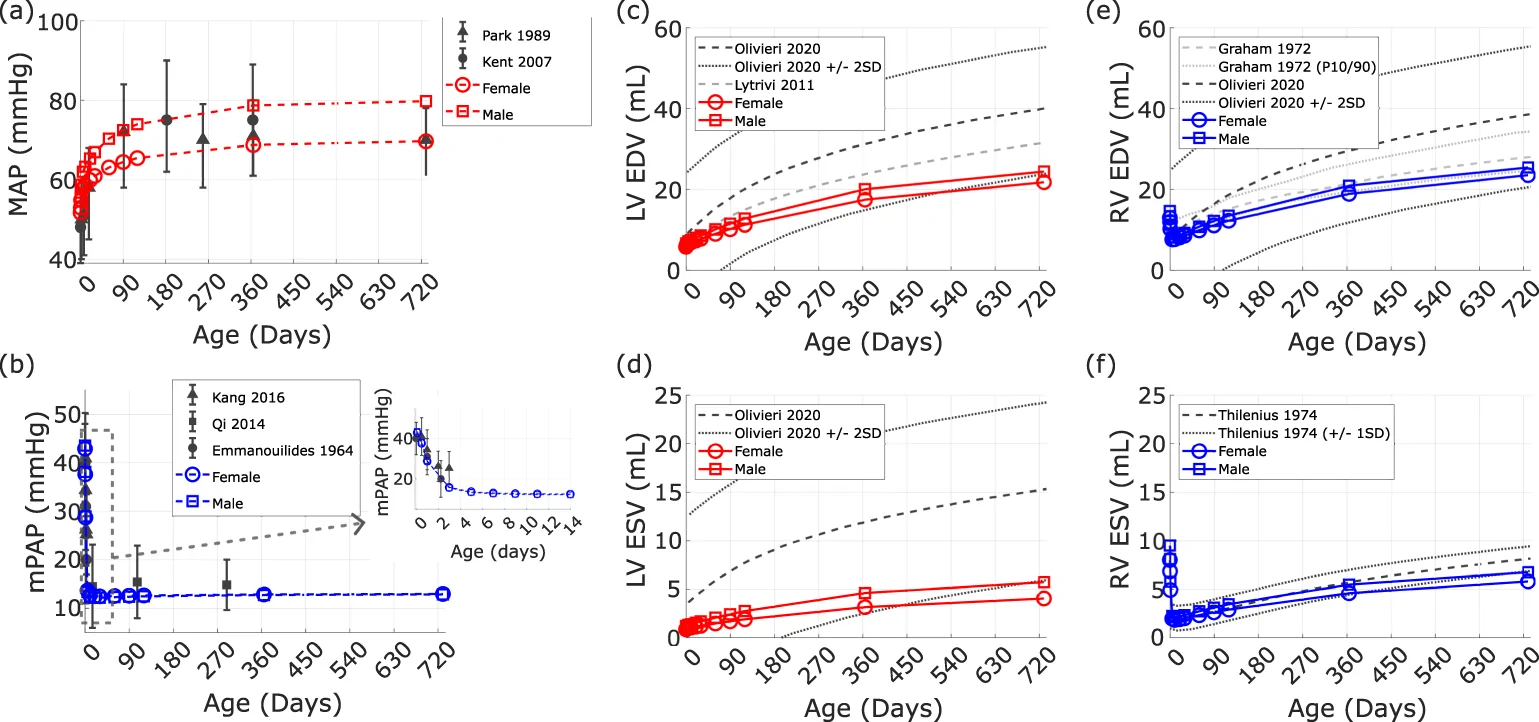
Changes in (a) MAP and (b) mPAP over the first 2 years of life in both females and males. Data from multiple studies [21–29] reproduced in the study by Hiebing *et al.* [8] are also presented. A subplot of mPAP during the first two weeks of life is also provided in (b). End-diastolic volumes (EDV, (c) and (d)) and end-systolic volumes (ESV, (e) and (f)) are presented over the same time frame as subplots (a) and (b). Data from multiple studies [21–29] reproduced in the study by Hiebing *et al.* [8] are also presented.

We compare our model results between a male adult and a male newborn (2 days of age) in figure 3. Female predictions are provided in figure S1 in the supplementary material. Adult simulations in figure 3a,b,c show physiologically consistent blood pressure predictions and realistic, biphasic tricuspid and mitral valve flow patterns [20]. Atrial pressure–volume loops do not capture the more complex ‘a’ and ‘v’ loops seen in typical LA and RA dynamics. Paediatric simulations show several differences from adult haemodynamics. Cardiac volumes are significantly smaller, consistent with smaller heart size. Equations (2.10) and (2.11) enforce a lower SVR and higher PVR with earlier age, resulting in a decreased LV systolic pressure and elevated RV systolic pressure, as shown in figure 3d. PA pulse pressure in figure 3e is higher, consistent with a small pulmonary vascular compliance. Flow simulations in figure 3f are similar in shape to the adult simulations in figure 3c, but with substantially smaller magnitudes. Heart rate is also faster in the paediatric case.

**Figure 3.**
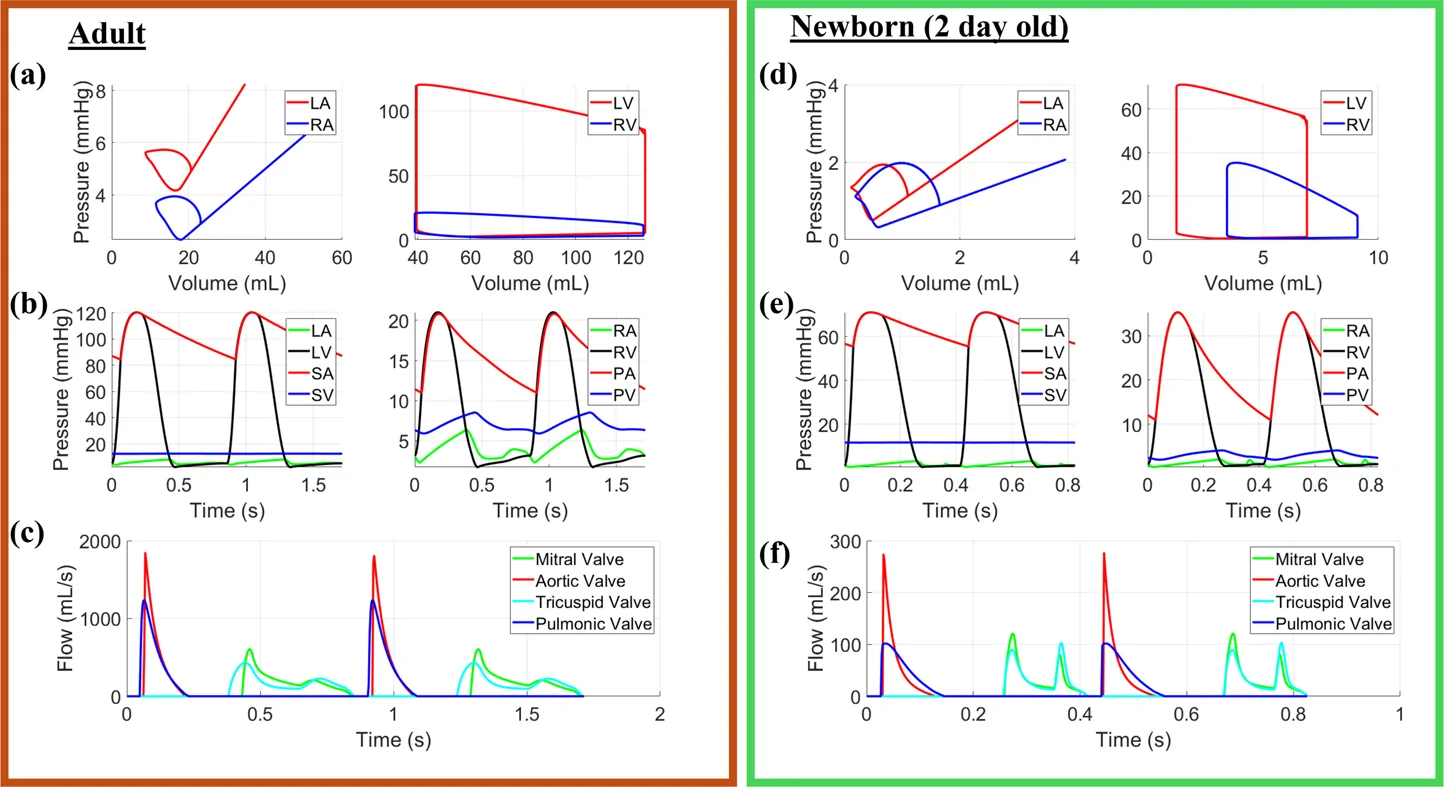
(a–c) Simulations in a typical adult, 70 kg male, including atrial and ventricular pressure–volume loops (a), vascular pressures (b), and valvular flows (c). (d–f) Simulations from the same model but with parametrizations based on allometric and maturation-based scaling laws to a 2-day-old. Note that pulmonary pressures are significantly higher, with smaller cardiac volumes. See figure 1 for abbreviations for the model infrastructure.

### 3.2. Simulating ventricular septal defect, growth and the effects of pulmonary vascular dysfunction

We simulate a 7-day-old female (figure 4a,c) and male physiology (figure 4b,d) across four cases: no VSD and small, medium or large VSD. The last three are parametrized by VSD diameters of 1, 3 and 6 mm, respectively. Haemodynamics are similar between no VSD and a small VSD across both sexes. Medium-sized VSDs increase RV pressures and slightly decrease LV pressures, with a small rightward shift in RV volumes shown in figure 4a,b. There is also an increase in LA pressure and volume, whereas RA haemodynamics minimally change. Large-sized VSDs amplify prior haemodynamic changes, with systolic pressures in the two ventricles approaching a similar magnitude. A large VSD increases RV EDV, while LV stroke volume increases along with LV EDV. There is a slight sex difference in RV EDV, with larger values in males.

**Figure 4.**
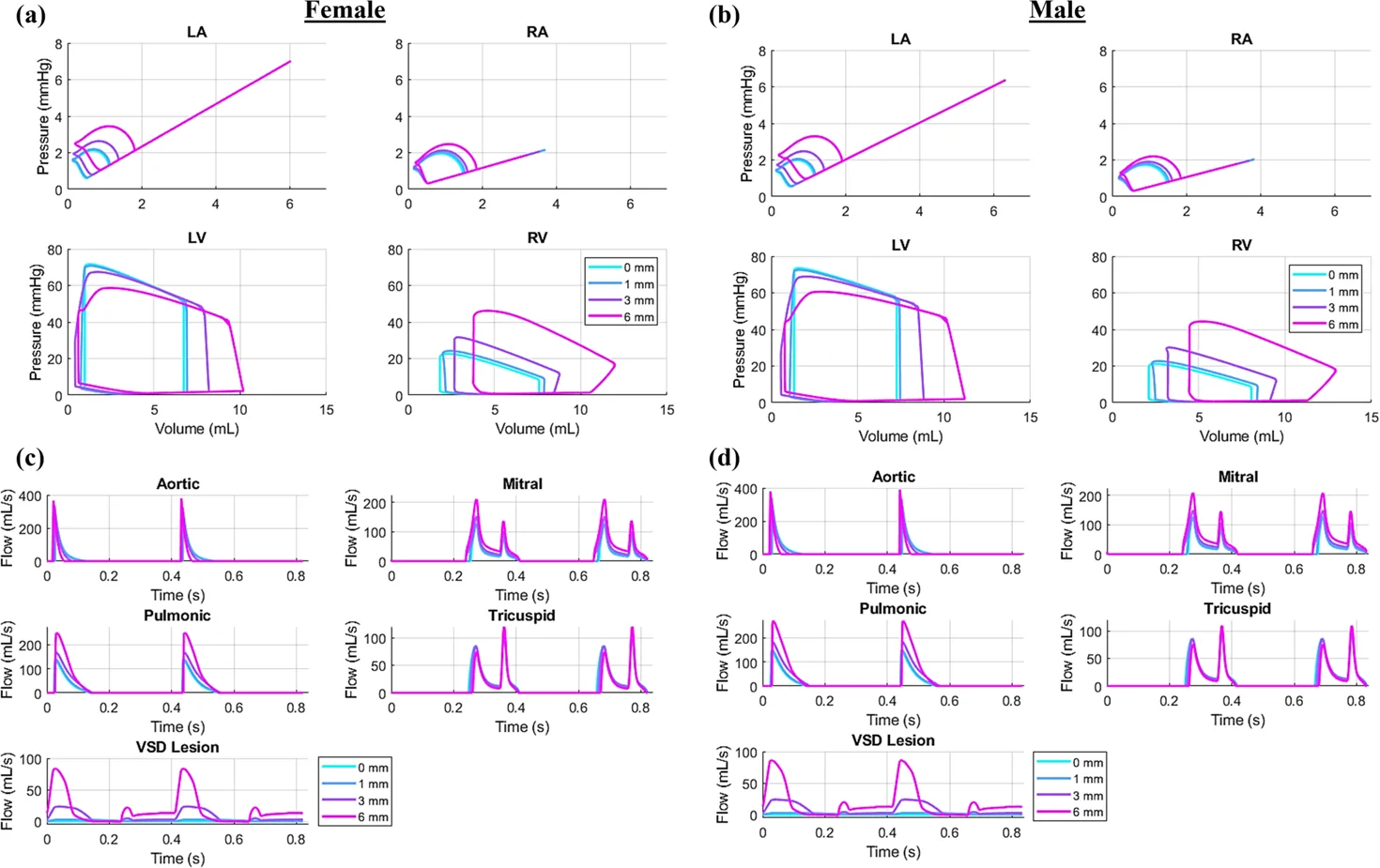
Simulations in a 7-day-old paediatric female (a,c) and male (b,d) subject with and without a VSD. Cardiac function (a,b) and valvular flows (c,d) are shown for each sex with no VSD, a small VSD, a medium VSD and a large VSD, corresponding to 0, 1, 3 and 6 mm in diameter, respectively. Subplots (c) and (d) include flow across the VSD, with positive flow indicated by left-to-right shunting.

Valvular and VSD flow are shown in figure 4c,d. Left-to-right shunts (indicated by positive VSD flow) increase with larger VSD sizes. Though tricuspid flows are similar, mitral valve flow is larger in magnitude for larger VSDs. Large VSDs also increase pulmonic valve flow and reduce the time during which the aortic valve is open, leading to larger total pulmonary flow relative to systemic flow (i.e. *Q_p_*/*Q_s_*).

We investigated how VSD size and PVR may impact simulated cardiac and haemodynamic metrics. Results in figure 5 show six simulated scalar metrics against different PVR magnitudes for a 30-day-old male subject with various VSD sizes. These include the *Q_p_*/*Q_s_* ratio, mPAP and cardiac work in the LV, RV, LA and RA. The PVR values are presented in Wood units (WU), which are equivalent to mmHg min l^−1^. Cardiac work was defined as the area within the pressure–volume loop and converted to Joules using the conversion factor 1 J = 7.5 × 10^3^ mmHg ml. The *Q_p_*/*Q_s_* magnitude is significantly higher in medium and large VSDs compared with small or no VSD. As PVR magnitude increases, we get a decrease in *Q_p_*/*Q_s_* such that at 30 WU, even large VSDs fall below the 1.5 ratio used clinically for assessing the haemodynamic effects of a VSD [11]. However, such values of PVR are quite high and represent a severe pathology. Larger PVR values increase mPAP, with pulmonary hypertensive conditions defined for mPAP > 20 (provided in figure 5b). The difference in mPAP between small and large VSDs is apparent for low PVR, but larger PVR values diminish this difference.

**Figure 5.**
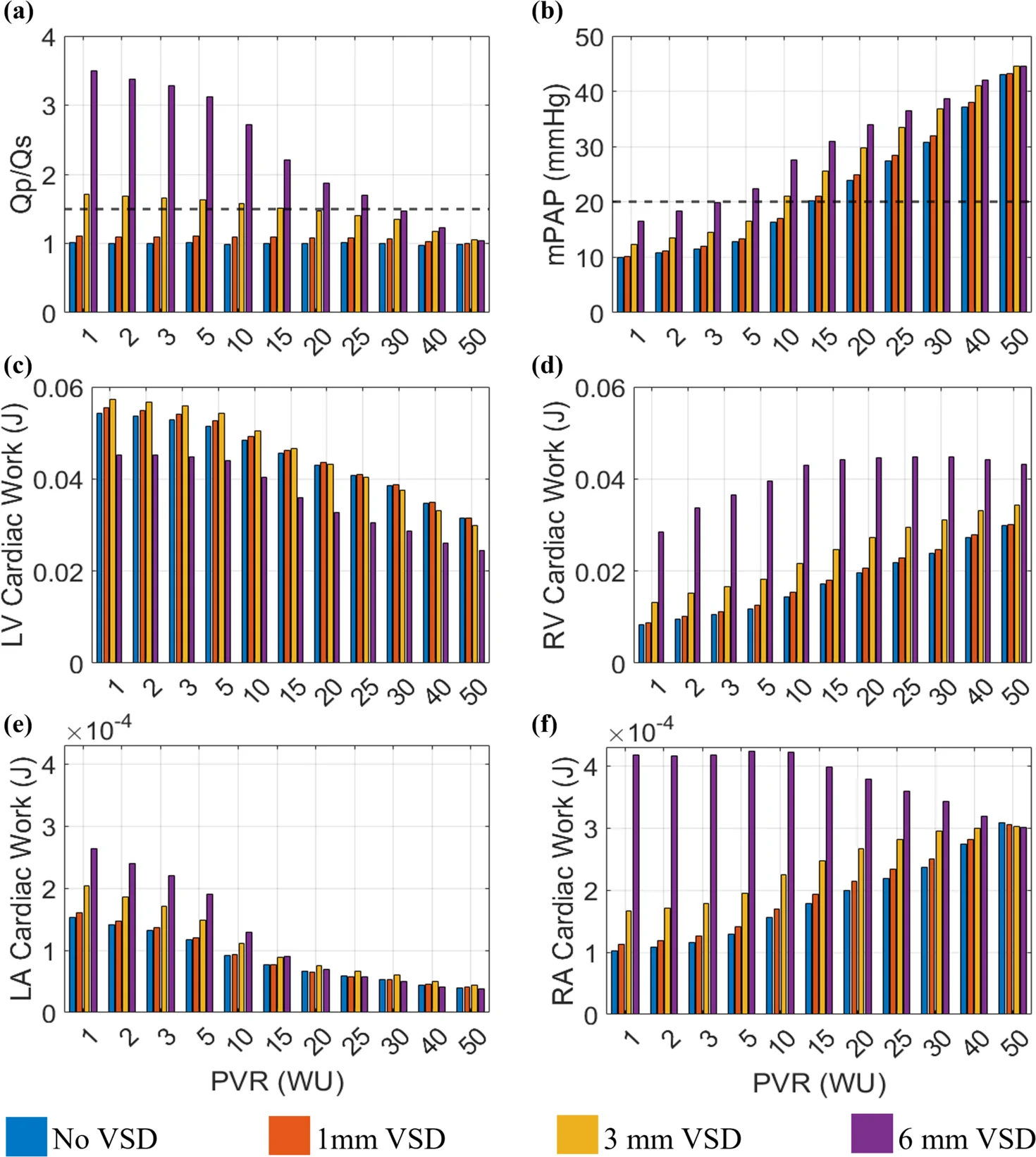
Metrics of haemodynamic and cardiac function for various VSD sizes (provided as diameter) and PVR magnitudes, presented in Wood units (WU), for a 30-day-old male subject. (a) Ratio of mean pulmonary flow to mean systemic flow (*Q_p_*/*Q_s_*), with the threshold of 1.5 provided. (b) Mean pulmonary arterial pressure (mPAP), with the threshold of 20 mmHg used to define pulmonary hypertension. (c–f) LV, RV, LA and RA cardiac work calculated from simulated pressure–volume loops.

Results in figure 5c–f show cardiac work in all four heart chambers. LV cardiac work is smallest in the case of a large VSD, consistent with the largest left-to-right shunt. Increasing PVR reduces LV cardiac work. The opposite holds for the RV, which has significantly higher cardiac work for a large defect. Increasing PVR causes a decrease in LV work and an increase in RV work. Work by the LA is highest for low PVR and a large VSD. Large VSDs increase RA work as well, but RA work decreases with higher PVR.

We simulated ageing using the allometric and maturation approaches in equations (2.9)–(2.11) across non-VSD and small-, medium- and large-sized VSDs. Figure 6 shows LV and RV pressure–volume loop simulations in a female subject. Both LV pressures and volumes tend to increase with age. The LV exhibits increased EDVs and stroke volumes with larger defect sizes. Systolic pressures in the LV at 2 years of age are noticeably lower with a large VSD than with no VSD. In the RV, we see elevated pressures in all VSD conditions across the first 3 days of life. There is a clear increase in EDV and ESP in the RV with larger VSDs at 3 days of age. There is an abnormal curve to the RV PV loop during isovolumic contraction, which is attributed to increased volume through the left-to-right shunt. Predictions at days 30, 60 and 90 show more subtle differences between no VSD and small- and medium-sized VSD predictions. The RV ESP is clearly elevated in medium- and large-sized VSDs, even though *R_p_* is the same.

**Figure 6.**
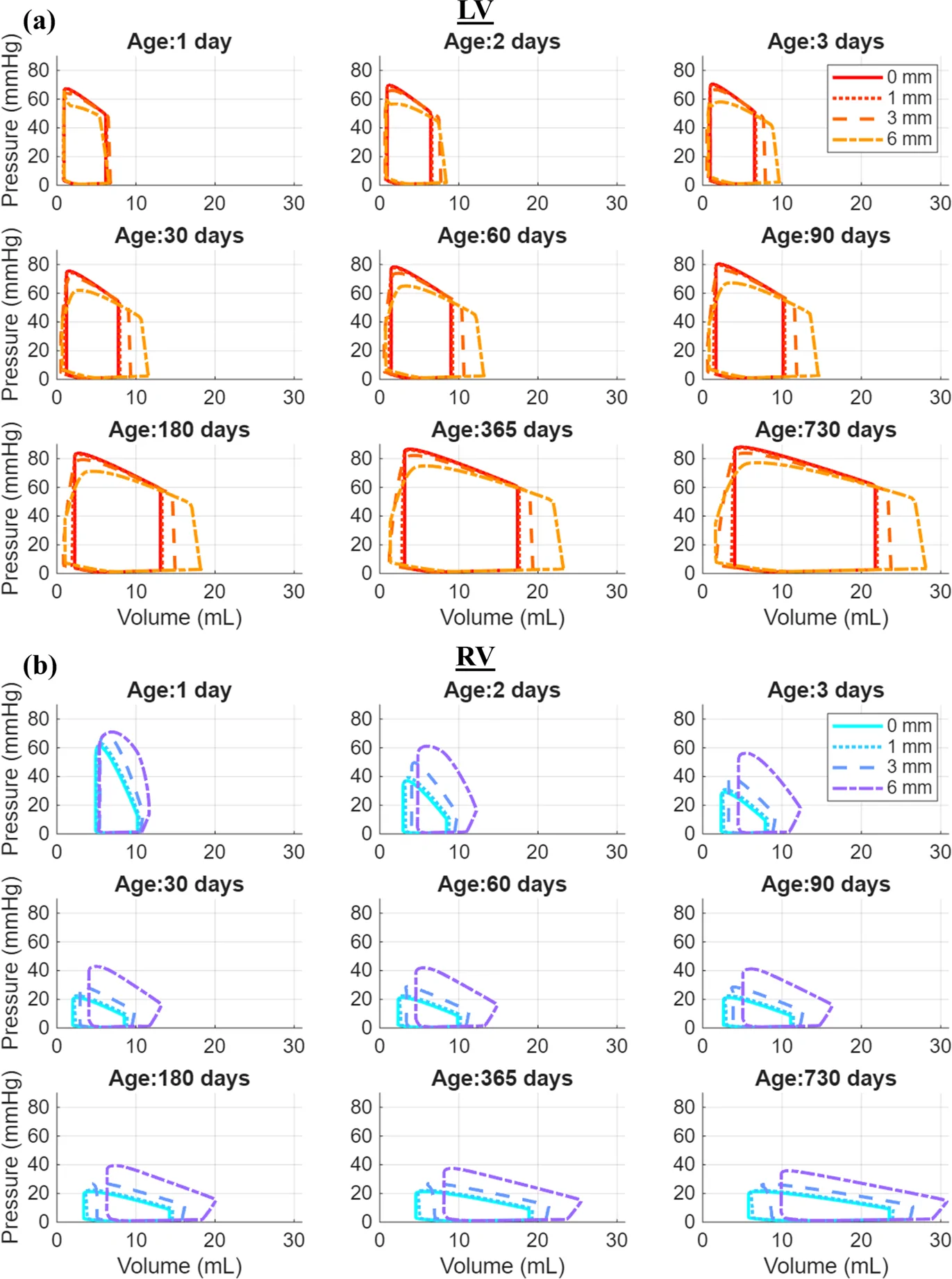
Ventricular pressure–volume loops as a function of age and VSD severity in a female. Parameters of the model are scaled using allometric and maturation-based approaches as described in the text. (a) Haemodynamics in the LV from 1 day to 2 years of age. VSD diameters used in the simulations are 0, 1, 3 and 6 mm in diameter. (b) Haemodynamics in the RV over the same time span.

We conducted a similar set of simulations in figure 6 but with different pulmonary vascular adaptation scenarios. The first set of simulations (figure 7a,b) use the maturation function to progress until day 30, at which time the value of *R_p_* (*t*) = *R_p_* (30) and *C*_pa_ (*t*) = *C*_pa_ (30). The second set of simulations (figure 7c,d) allow pulmonary adaptation until 3 days of age. In the former case (figure 7a,b), we see relatively little change in the LV compared with results in figure 6. In contrast, the RV undergoes a relatively large drop in pressure from 1 day to 30 days of age but then experiences an increase in pressure magnitudes at later age points. This is more prominent for the large-sized VSD, where there is an accompanying increase in RV EDV with larger defects. For the second set of simulations, we see that LV EDV is similar with all VSD sizes, with only minor changes in pressure (figure 7c). The RV predictions (figure 7d) show an elevated ESP across all four time points, with a slight decrease in pressure for small- and medium-sized VSDs at 30 days of age. The RV EDV values for no-VSD and the three VSD sizes are similar when PVR remains high.

**Figure 7.**
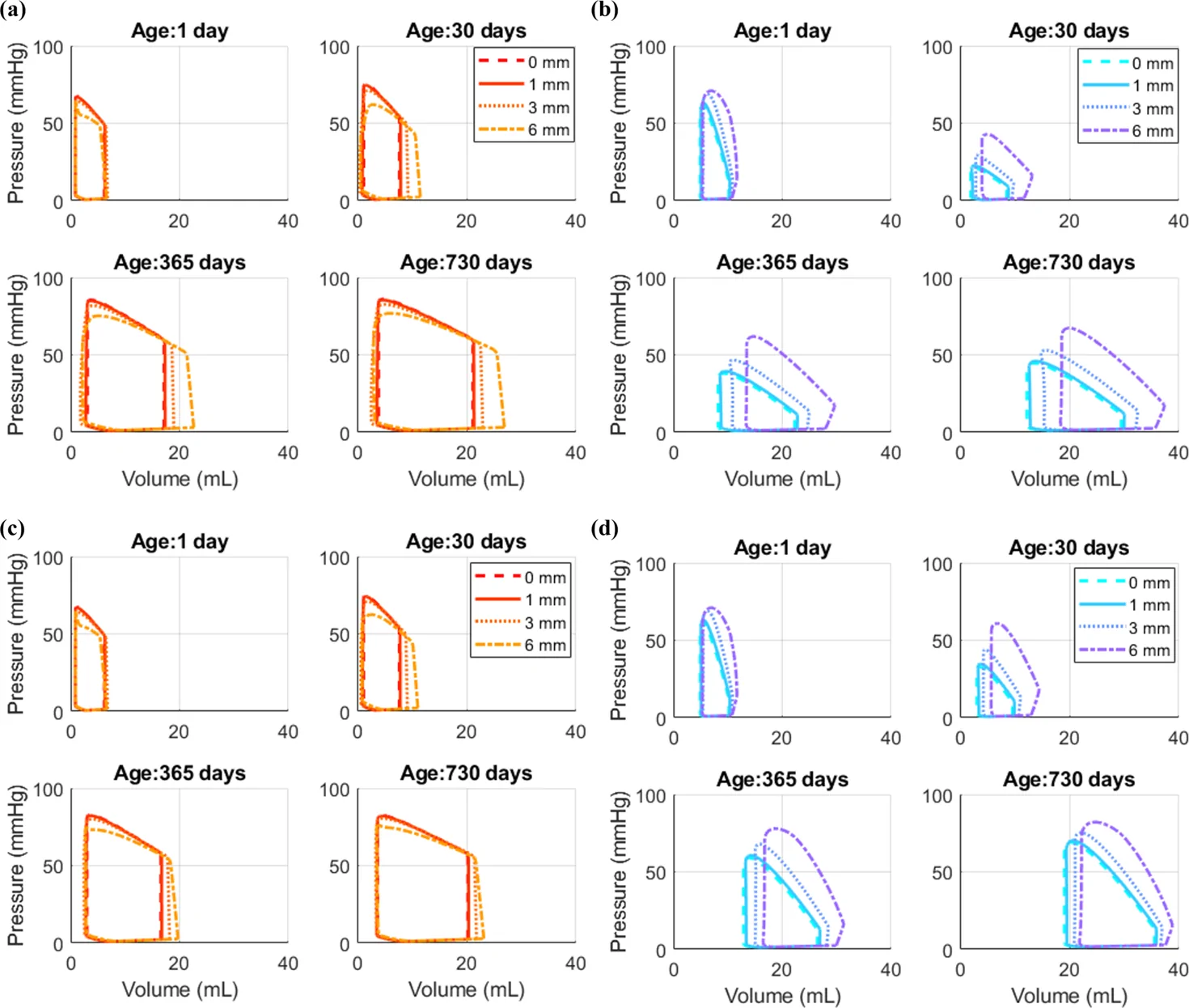
Ventricular pressure–volume loops as a function of age and VSD severity with different pulmonary vascular conditions in a female. All parameters of the model are scaled as described in the text except for *R_p_* and *C*_pa_. (a and b) Haemodynamics in the LV and RV from 1 day to 2 years of age under the assumption that *R_p_* and *C*_pa_ change until 30 days of age. (c and d) Haemodynamics in the LV and RV from 1 day to 2 years of age under the assumption that *R_p_* and *C*_pa_ are fixed at the value obtained at 3 days of age.

### 3.3. Sampling-based uncertainty propagation for age-dependent haemodynamics

Finally, we consider how uncertainties in the maturation parameters of equations (2.10) and (2.11) and total blood volume may change the trajectory of haemodynamics as a function of age. Arterial resistance and compliance trajectories are presented in figure 8, which shows larger uncertainty in *R_p_*, *R_s_* and *C*_pa_ during the initial stages of the maturation curve. In contrast, *C*_sa_ has relatively constant uncertainty throughout 2 years of life. We subsequently propagated these uncertainties through the model to provide individual trajectories up to 2 years of age for each curve presented in figure 8. We did this in small-, medium- and large-sized VSDs. Figure 9a–c shows uncertainty in LV pressure–volume loops with each VSD size and across 2 years of age. We see that LV pressures decrease with VSD size at early-age points. This is maintained throughout the rest of the 2-year period. However, EDV and stroke volume are higher at 2 years of age in large-sized VSDs (figure 9c), with larger pressure–volume loop area. In general, the uncertainties in cardiac dynamics are larger with early age, with relatively large uncertainty at later ages as well for medium- and large-sized VSDs. Simulations in the RV (figure 9d–f) also show larger uncertainty at early ages. Interestingly, the small-sized VSD simulations (figure 9d) at 1 day of age have a nearly identical uncertainty to medium- and large-sized VSDs. The average RV pressure–volume loop shape at 1 day of age is distinct between VSD sizes, with a more triangular loop in the small-sized VSD case that becomes rounded for medium- and large-sized VSDs. There is a large reduction in pressure magnitudes and their uncertainty at 5 days of age, with less uncertainty as age increases. As expected, later time points suggest elevated RV pressure for the larger VSD sizes. We also see larger ESV, EDVs and stroke volume for the large VSD simulations.

**Figure 8.**
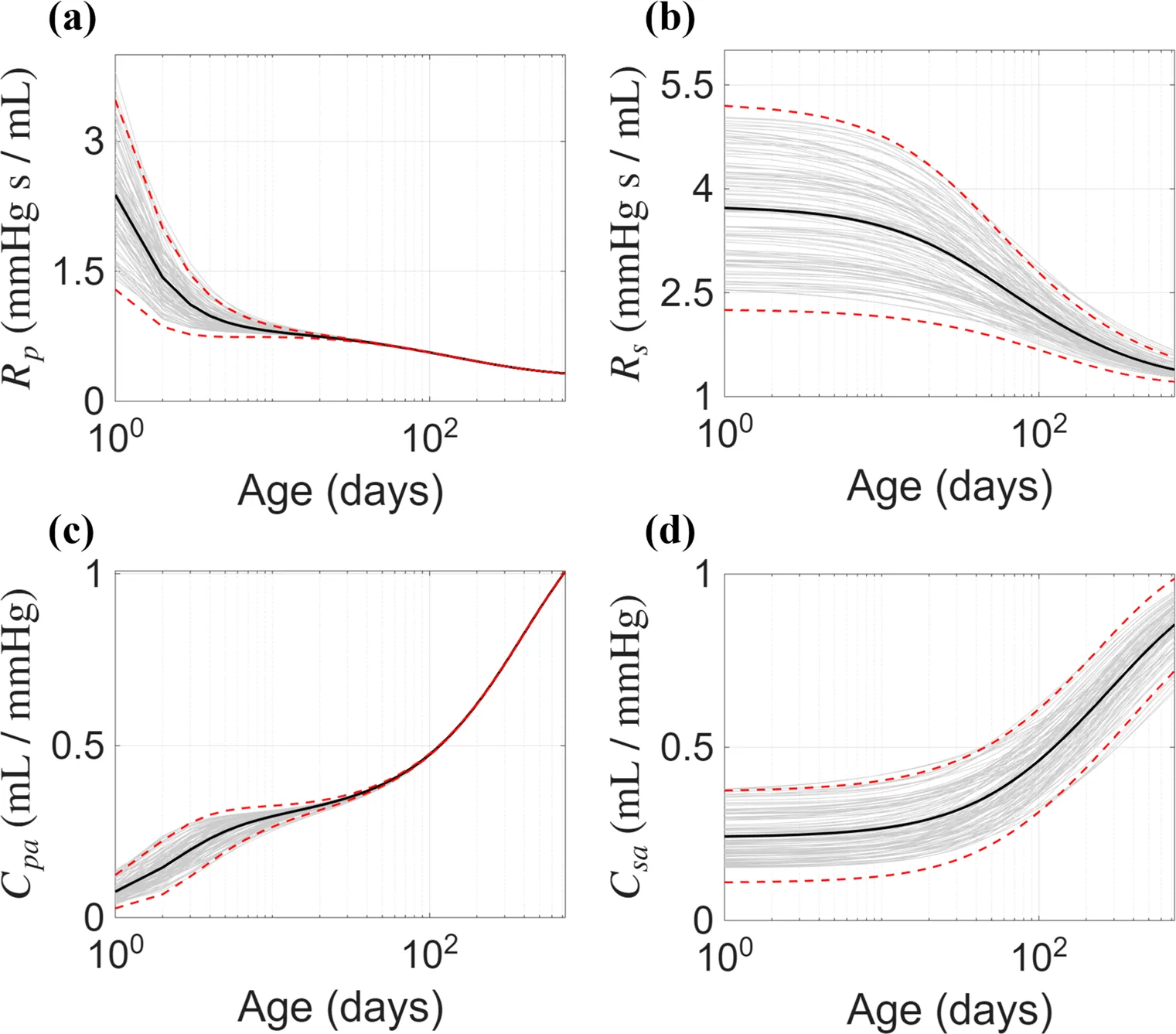
Variability in (a) pulmonary vascular resistance (*R_p_*), (b) systemic vascular resistance (*R_s_*), (c) pulmonary vascular compliance (*C*_pa_) and (d) systemic vascular compliance (*C*_sa_) when sampling maturation parameters in a female. Note that age on the x-axis is provided on a log-scale for better interpretability. Grey lines indicate 100 realizations, while the solid black lines and dashed red lines indicate the average and two standard deviations from the average, respectively.

**Figure 9.**
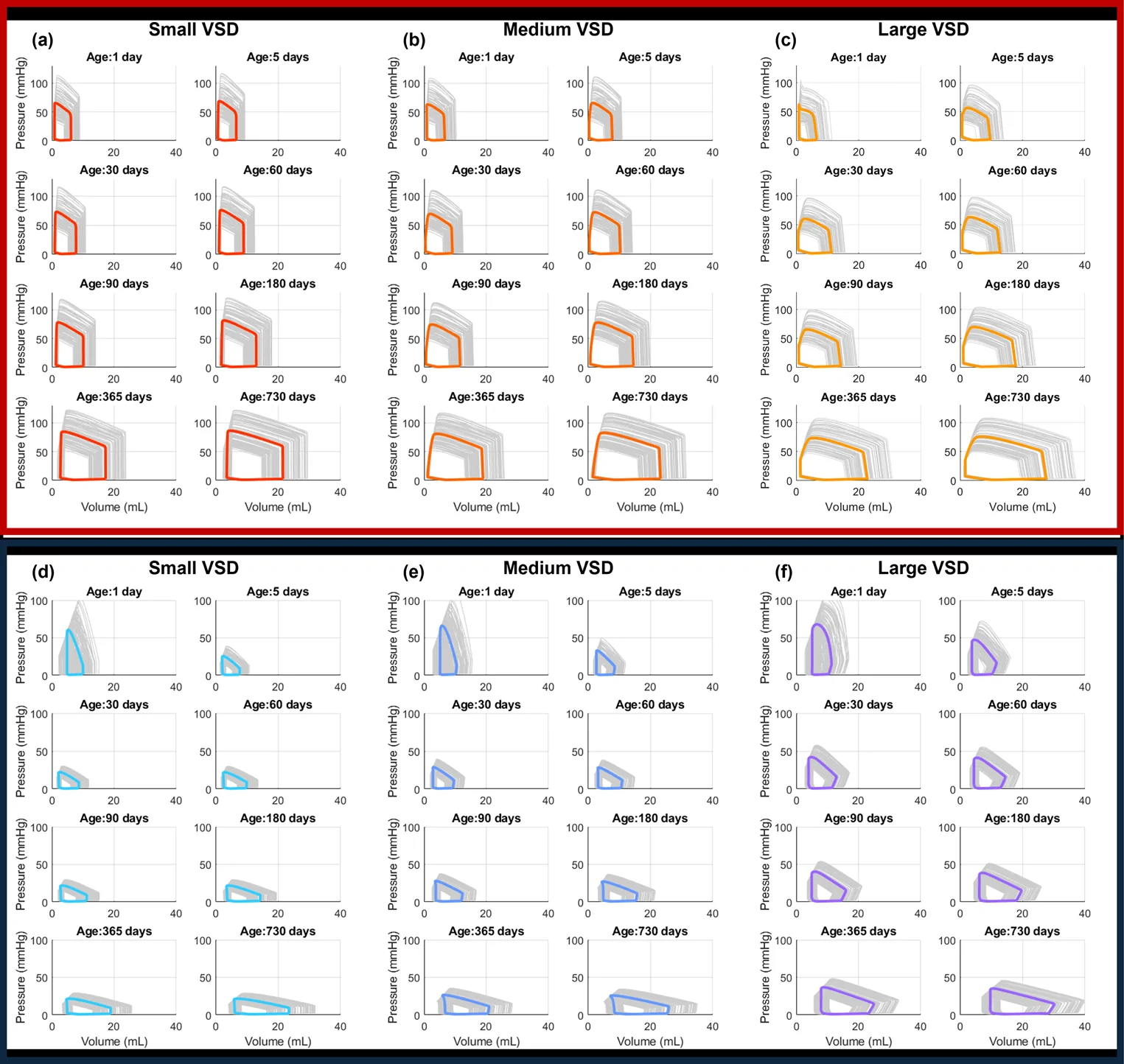
Variability in LV (a–c) and RV (d–f) pressure–volume loops for small (a and d), medium (b and e) and large (c and f) VSD sizes, corresponding to 1, 3 and 6 mm diameters in a female. Simulations use the pulmonary and systemic vascular parameter curves from figure 8, as well as uncertain total blood volume. Grey lines indicate 100 realizations, while the solid-coloured lines indicate the average.

We examined how VSD flow magnitudes (figure 10) and the scalar metrics MAP, mPAP and *Q_p_*/*Q_s_* (figure 11) varied with uncertainty. VSD flow consistently shows higher relative uncertainty at early ages in comparison with later age points. All VSD sizes transition from having shunt flow in both directions to predominately left-to-right shunt flow (indicated by positive flow magnitudes) at 5 days of age. Large-sized VSDs still exhibit some bidirectional shunt flow at later ages, which is attributed to the uncertainty in *R_p_*. Small- and medium-sized VSDs have relatively constant left-to-right shunt flow magnitudes with increasing age.

**Figure 10.**
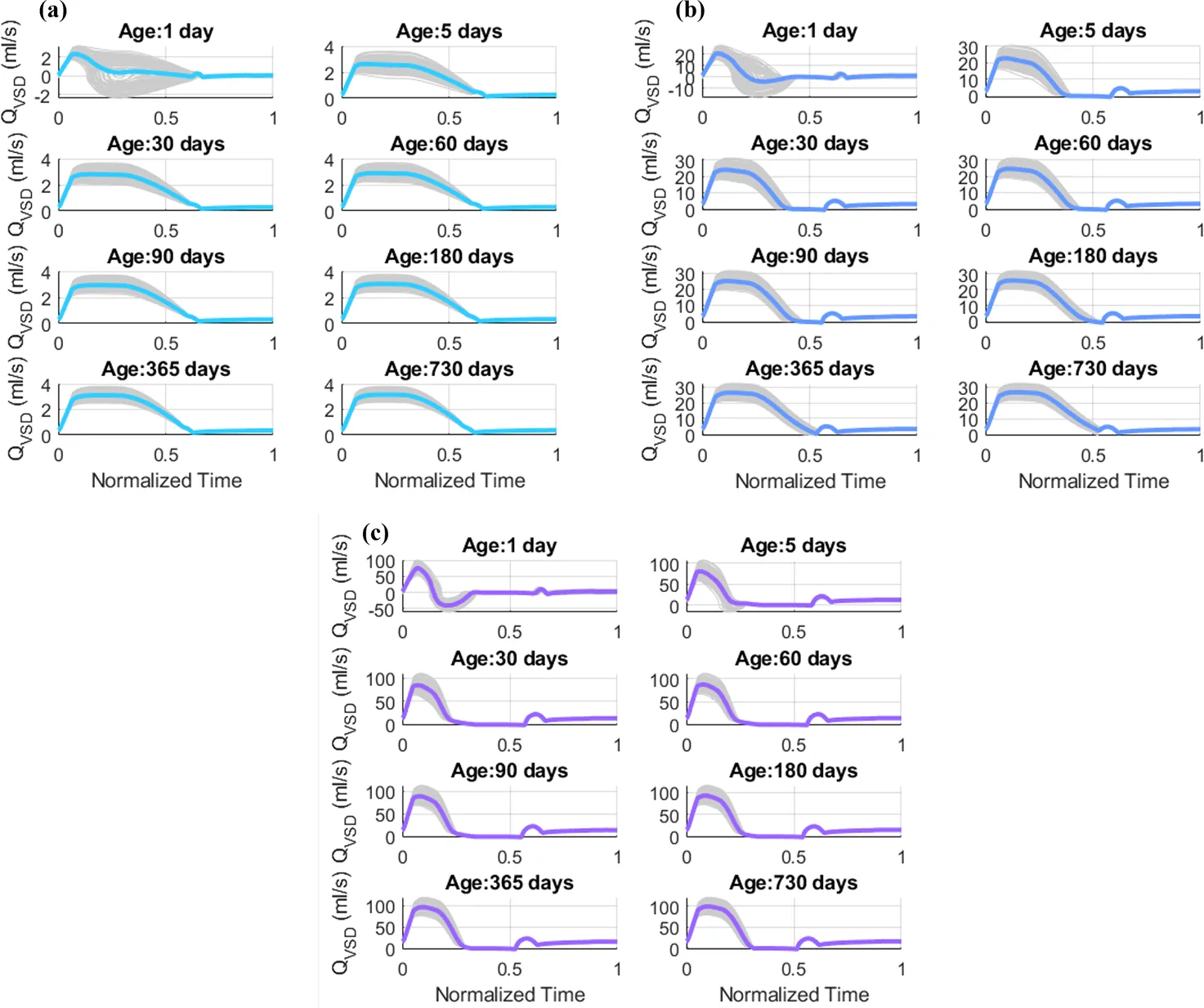
Variability in female VSD flow for (a) small, (b) medium and (c) large VSD sizes, corresponding to 1, 3 and 6 mm diameters. Y-axes provide different magnitudes for ease of interpretability. Simulations use the pulmonary and systemic vascular parameter curves provided in figure 8, as well as uncertain total blood volume. Grey lines indicate 100 realizations, while the solid-coloured lines indicate the average.

**Figure 11.**
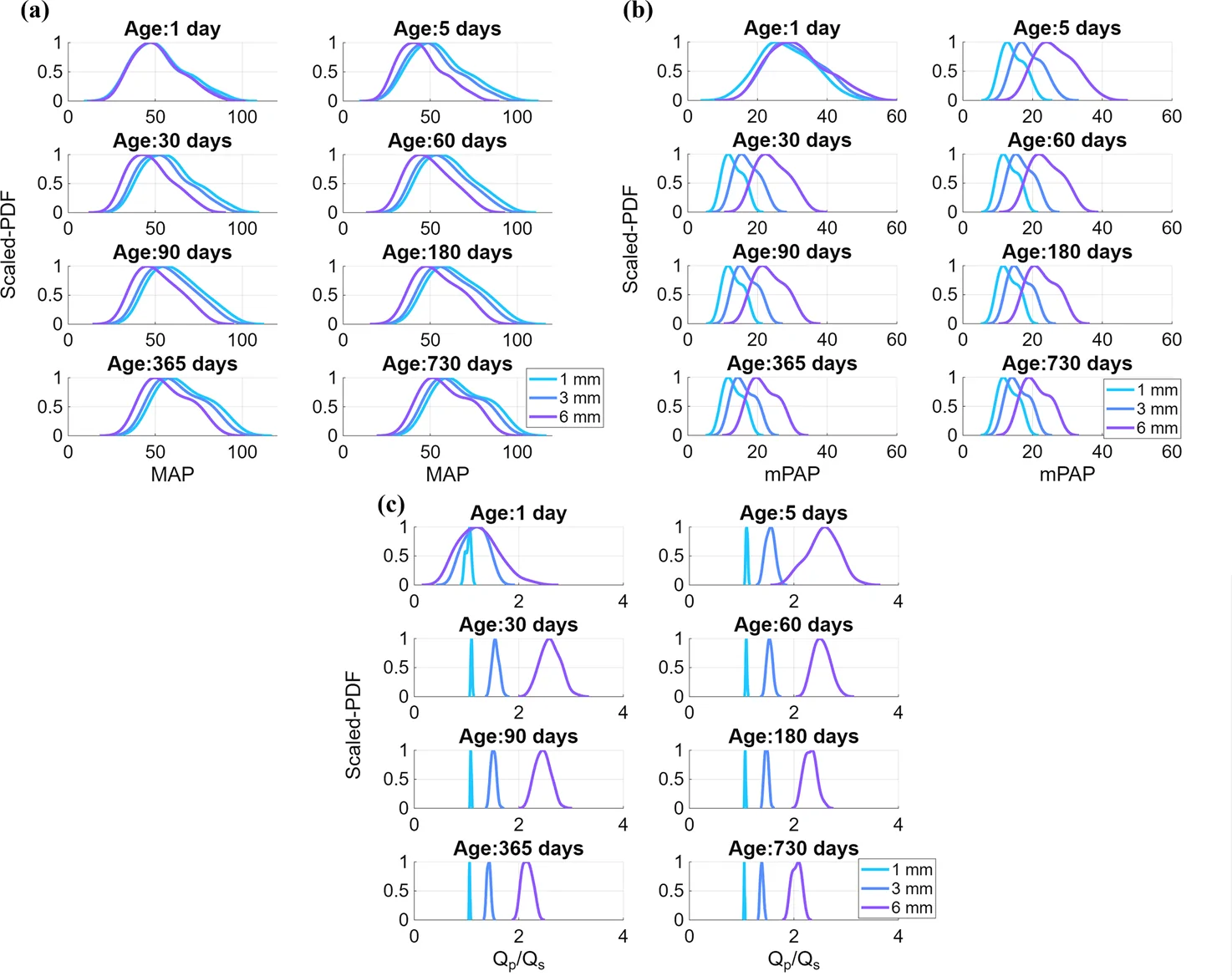
Variability in female (a) mean systemic arterial pressure (MAP), (b) mean pulmonary arterial pressure (mPAP) and (c) the pulmonary-to-systemic flow ratio (*Q_p_*/*Q_s_*) as a function of age and VSD size. We use scaled-probability density functions (PDFs), which are scaled such that the mode of each PDF is 1, for the sake of interpretability. The width of the distribution indicates uncertainty in the estimate, and the y-axis provides the magnitude of the quantity. Simulations use the pulmonary and systemic vascular parameter curves and uncertain blood volumes from before.

The scalar metrics provided in figure 11 show the scaled-probability density functions (PDFs) for MAP, mPAP and *Q_p_*/*Q_s_*, where the PDF modes have been scaled to one for comparison. We see MAP values are nearly identical across VSD size at 1 day of age, with larger differences between the VSD sizes occurring at later time points. MAP values have much larger uncertainty compared with mPAP, which is attributed to the more gradual variability in *R_s_* and *C*_sa_, as shown in figure 8. The values of mPAP (figure 11b) are nearly identical across VSD sizes at 1 day of age but shift to distinct PDFs at 5 days of age. Larger VSDs have a rightward shift in their PDF reflecting increased mPAP values. The variance of mPAP is similar in all three VSD sizes, though large-sized VSDs do lead to slightly larger variability. The results for the *Q_p_*/*Q_s_* ratio (figure 11c) illustrate more gradual changes in haemodynamics with age. The modes for all three PDFs overlap at 1 day of age, but the variance is larger for large-sized VSDs. This holds true through all age points, with the large-sized VSD having a variance nearly double that of the medium-sized VSD. As expected, small-sized VSDs have a *Q_p_*/*Q_s_* close to 1, whereas medium- and large-sized VSDs have *Q_p_*/*Q_s_* values in the ranges [1.2, 1.5] and [1.8, 2.3], respectively.

## 4. Discussion

We simulated cardiovascular function across the first two years of life using an eight-compartment, lumped-parameter model of the four heart chambers and major circulations. We usea previously developed method for allometric and nonlinear scaling of model parameters with age to investigate the effects of VSD on haemodynamics. Larger defects increase flow across the VSD and pulmonary flow, increasing right-sided loading after the early neonatal period. Impaired PVR adaptation (modelled here as an arrested decline) sustains elevated RV pressures and higher mPAP. Uncertainty analysis on the scaling laws for PVR and SVR shows substantial uncertainty in early-age points, showing a significant impact of vascular resistance on early-age haemodynamics.

### 4.1. Simulating growth in a simple compartment model

Hiebing *et al.* [8] provided a foundational methodology for scaling PVR and SVR to simulate cardiovascular adaptation in newborns. A prior study by Munneke *et al.* [9] simulated the transition from fetal to neonatal circulations, including flow through the foramen ovale and ductus arteriosus, with a focus on oxygen saturation levels. Chen *et al.* [7] recently simulated haemodynamics in neonates with borderline LV, a condition where neonates are born with an underdeveloped LV that is on the border of being diagnosed as hypoplastic left heart syndrome. Chen *et al.* also used the adaptation strategy of Hiebing *et al.* to scale model parameters for the circulation and showed agreement with clinical data. The scaling laws for our eight-compartment model provide similar physiological outputs over 2 years of age, as shown in figure 2. Predictions of MAP and mPAP are within the bounds of data collected in physiologically normal paediatric subjects across multiple measurement modalities [21–29]. Our results underperform in predicting ESVs, where we tend to have smaller heart values near the minimum bounds from prior data. However, the framework does capture the rapid drop in mPAP and a previously documented reduction in RV volumes immediately after birth with the drop in PVR [20].

Our adult simulations, used for scaling to paediatrics, are within the range of previously recorded normotensive individuals [17,32–34]. We simulated sex differences by providing slightly lower systemic and pulmonary pressures, higher heart rate and smaller cardiac volumes in the female sex [20]. Our simulations of neonate haemodynamics are similar to adult haemodynamics in shape but are drastically different in magnitude. Neonates have lower cardiac volumes, higher pulmonary pressures and lower systemic pressures, consistent with our findings [9,23,24]. Neonatal PA pulse pressure is higher than the adult simulations owing to lower *C*_pa_ values, and simulated RV volumes are slightly larger than LV volumes. Our paediatric simulations of mitral valve flows show an *E*/*a* ratio, computed from the early and late flows into the LV, greater than 1, which increases with later ages. In contrast, the tricuspid *E*/*a* ratio is less than one in paediatrics and then increases with later ages. This is consistent with prior echocardiography data [35] which showed that *E*/*a* increases with age and can be close to or less than one in paediatric subjects.

### 4.2. Haemodynamic changes with ventricular septal defect

There are drastic changes in haemodynamic parameters at birth. These effects are modulated when a VSD is present [11,35]. The left-to-right shunt across the septum increases pulmonary vascular flows and changes haemodynamic loading [11,13]. Two key factors in determining left-to-right shunt are the VSD size and PVR, both contributing to the flow through the lesion. Our simulation results in figure 4 assess the effects of VSD size at 7 days of age and show larger left-to-right flow magnitude with larger VSD sizes. Pressure–volume loops show increased EDV in both ventricles and the LA, with little change in RA volumes. Medium- and large-sized VSDs often induce increased LA and LV preload and dilation [14,36], which is consistent with our simulations. The larger defect sizes increase LA pressure and LV stroke volume also. Our simulations also show a rightward shift in the RV, reflecting an increased EDV with increased RV pressures. While pressure increases are expected [36], increased RV EDV is uncommon. However, this can occur when PVR remains relatively low [36], which is consistent with our results since PVR drops quickly and is relatively low at 7 days of age. Moreover, we do not explicitly include RV remodelling, and prior studies have shown that significant left-to-right shunts can lead to RV hypertrophy [35,37]. Our simulations show abnormal RV isovolumic contraction with a VSD, as RV volume increases during contraction owing to the left-to-right shunt. Larger VSDs also increased the *E*/*a* value of the mitral valve, whereas the tricuspid valve flow is unaffected.

The interactions between PVR and VSD size dictate the haemodynamic state of a subject [7,29,36]. Figure 5 illustrates this interaction, as haemodynamic metrics at 30 days of age change drastically with VSD size and PVR values. The value of *Q_p_*/*Q_s_* is valuable in the clinical management of VSD [11,35,36], often used to determine the significance of VSD. For instance, larger *Q_p_*/*Q_s_* values have been associated with lower cerebral oxygen saturation [38]. We show that low to normal PVR can lead to a *Q_p_*/*Q_s_* > 1.5 in medium- and large-sized defects, but that higher PVR can reduce this ratio. We also quantify cardiac work, which provides insight into the effort needed by each cardiac chamber [39,40]. Given the difficulty of measuring pressure–volume loops in neonates, relatively few studies have documented these metrics. One study is Yang *et al.* [41], who compared clinical pressure–volume loops using pressure signals from right heart catheterization and volume estimates from magnetic resonance imaging in paediatric subjects with pulmonary hypertension. The authors showed that RV stroke work correlated with increased PVR over multiple time points in their patient population, similar to our findings. Works by Chowdhury *et al.* [42–44] used pressure–volume loops in the LV to assess contractility and diastolic stiffness in paediatric subjects, with results showing negligible differences in LV stroke work between those requiring and not requiring heart transplantation. Our results show that LV and RV stroke work move in opposite directions: larger VSDs and higher PVR reduce LV stroke work, while RV stroke work increases dramatically for large-sized VSDs and gradually with higher PVR. A large VSD with low or normal PVR has a high *Q_p_*/*Q_s_*, increasing RV volumes and stroke work. Though higher PVR decreases *Q_p_*/*Q_s_*, this elevates RV pressure and causes a steady increase in RV stroke work. These results suggest that the presence of a medium to large VSD drastically increases work done by the RV, and increased PVR owing to limited pulmonary vascular adaptation would increase this workload. LA and RA stroke work are relatively novel and rarely used metrics but may provide insight into cardiac function in this subject group [43,44]. The use of stroke work in general for assessing neonate function is uncommon but may provide insights beyond current diagnostics [40,45].

### 4.3. Effects of ventricular septal defect across age

Detection and diagnosis of VSD by echocardiography is critical at the early stages of life. A computational model can provide significant additional insight if it can simulate haemodynamic trajectories with adaptation at later ages. In the case of medium-sized VSDs, the decision to operate can be riddled with uncertainty [11,12,16], providing an avenue for assistance by computational modelling. The LV pressure–volume loops in figure 6a show the natural changes in haemodynamics with and without a VSD. Both LV EDV and ESV increase with age, indicative of natural weight gain and increased blood volumes, along with increased contractility. LV pressures naturally rise with increased SVR and reduced systemic vascular compliance, consistent with prior findings [9,46]. In the presence of a medium- or large-sized VSD, we observe larger LV EDV, with a prominent increase in LV stroke volume at the later age points. This increase in stroke volume coincides with reduced LV pressure and highlights the model's ability to reproduce LV volume overload with larger VSDs [11,12]. The RV undergoes a rapid transition during the initial stages of life in the absence of a VSD, with significantly larger systolic pressures at 1 day of age, followed by a drop in pressure magnitudes and eventual increase in blood volumes without the presence of a VSD. Small-sized VSDs cause little to no change in this trajectory. However, medium- and large-sized VSDs cause a delayed decrease in RV pressures, in addition to a rightward shift indicative of higher EDVs. The RV continues to have higher ESPs at the later age points, suggesting that a large-sized VSD in the absence of elevated PVR can still lead to pulmonary hypertensive conditions. Larger RV EDVs suggest dilation of the chamber, which is not a hallmark of untreated VSDs, in contrast to LV and LA dilation [35,37]. We attribute this discrepancy in part to our lack of explicit mechanisms for cardiovascular remodelling, which has been considered in several other studies focusing on cardiac [8] or vascular [29,47] adaptation. We found that either doubling *E*_min_ in the RV, reflecting an increased RV stiffness with hypertrophy, or decreasing SV compliance, *C*_SV_, by 25% to increase volume to the systemic circulation, reduced RV volume overload in our simulations. This result, provided in the supplementary material, supports the role of cardiac and vascular adaptation in the progression of untreated VSDs and should be investigated further.

 Pulmonary vascular adaptation is a major factor in VSD management. We considered stunted adaptation, where pulmonary vascular parameters were fixed at their 30-day or 3-day values. The former, shown in figure 7a,b, leads to increased stroke volume in the LV with all VSD cases, as was shown in figure 6. For the RV, we see biphasic behaviour. From 1 day to 30 days of age, we see a reduction in RV ESP and a mild increase in RV EDV for the large-sized VSDs. Then, during stunted adaptation, we see an increase in RV ESP at 1 year of age in all scenarios, with an even larger increase in RV pressures and EDV at 2 years of age for the large-sized VSD. A similar trend is seen in figure 7c,d when pulmonary vascular parameters only adapt until 3 days of age. The LV undergoes a smaller increase in EDV, while the RV shows a significantly higher ESP at 30 days of age. This holds for the non-VSD and all VSD sizes. The biphasic fluctuation in RV pressure which may occur in subjects with congenital heart defects with stunted pulmonary vascular adaptation [48–50] may lead to delayed recognition of pulmonary vascular disease in these subjects.

### 4.4. Effects of pulmonary and systemic arterial parameters

Given that the adaptation curves from Hiebing *et al.* [8] were constructed based on data from multiple studies, we investigated how uncertainties in the growth parameters contribute to differences in haemodynamics for our model framework. This is similar to a sensitivity analysis, which eight-compartment models have been subject to in prior studies [17,51,52]. Most studies, though, focus on a single time point without adaptation. One exception is the study by Jones and Oomen [53], which considered a compartment-level model with biventricular interaction and cardiac growth and remodelling. The authors calibrated and validated cardiac remodelling trajectories, under uncertainty, to data from an animal model of mitral valve regurgitation. Otherwise, uncertainty in adaptation rules has had limited investigation from a sensitivity or uncertainty analysis perspective.

Like Jones and Oomen, we sampled parameters describing adaptation and propagated these uncertainties over multiple time points. This provides us with more of a ‘population’ level view of our simulated haemodynamics. As shown in figure 8, uncertainty in the vascular resistance and compliance parameters is much larger at younger ages. However, we do not see a corresponding reduction in uncertainty at later age points in our simulated outputs, as shown in figures 9–11. In particular, the LV pressures in figure 9 have similar uncertainty across all age points, while LV volume uncertainty appears larger at later age points. This is attributed to our inclusion of total blood volume as a random variable and reflects the natural variability in blood volumes owing to weight gain and heterogeneous development in children. Interestingly, we see that LV pressure uncertainty in medium- and large-sized VSDs (figure 9b,c) is smaller than that in small-sized VSDs (figure 9a), while the converse is true about volume variability. In contrast to the LV, pressure variability in the RV is much larger at 1 day of age than at later time points, though there are still substantial fluctuations in volumes at 2 years of age. The interactions between VSD size, haemodynamic parameters and total blood volume contribute to the severity of VSD-affected congenital heart defects [36]. The larger uncertainty at early-age points coincides with our understanding of VSD diagnosis: the size of the VSD and subject-specific parameters of the neonate (e.g. elevated PVR and low weight gain [14,16]) interact to determine the haemodynamic loading of the heart. Our study also shows that both LV and RV volumes can vary significantly at later age points with the presence of a medium- or large-sized VSD. Volume overload is a risk factor in the development of heart failure in children with non-restrictive VSDs [14,16], consistent with our predictions of elevated cardiac volumes in figure 9.

Flow across the VSD, shown in figure 10, again supports the claim that earlier age points have relatively higher uncertainty. Flow across a VSD can be quantified by echocardiogram but is relatively hard to measure exactly [11,35,36]. In general, the larger the defect, the greater the magnitude of the left-to-right shunt. At the initial age point, we see nearly equal magnitude flows from left-to-right and from right-to-left. This coincides with a larger PVR and similar magnitudes of LV and RV pressure, thus allowing flow in both directions. This quickly dissipates at five days of age as PVR begins to fall. Our systems-level results in figure 11 show that systemic haemodynamics are relatively consistent across age points and VSD size. As expected, higher PA pressures track with large-sized VSDs. The uncertainty in these quantities tends to decrease with age as well. The *Q_p_*/*Q_s_* values also vary with age and VSD size. As we saw in figure 10, the *Q_p_*/*Q_s_* values are relatively similar in their average value but have larger uncertainty for larger VSD sizes. At 5 days of age, when PVR has dropped, we achieved a distinct shift in the PDFs for *Q_p_*/*Q_s_*, with medium- and large-sized VSDs being centred around 1.5 and 2, respectively. These results are again consistent with guidelines on managing VSD, with *Q_p_*/*Q_s_* values in the range of 1.6–2.8 serving as clinical cut-offs [11,54,55]. Thus, our results show that medium- and large-sized defects reproduce *Q_p_*/*Q_s_* ratios that are consistent with those seen in the clinical management of VSD.

### 4.5. Future clinical relevance

VSDs are the most common congenital heart defect; hence, studying the interactions between haemodynamic conditions, VSD size and subject size has the potential to affect a large population. We show how lumped-parameter models can incorporate both (i) age- and weight-dependent adaptation through model parameters and (ii) various VSD sizes through a simple VSD size-resistance relationship in the model. These two contributions provide the necessary groundwork for generating subject-specific models that can be calibrated to clinical echocardiography data [6,7]. This would provide a clinically significant pipeline for monitoring medium-sized VSDs, as these defects may either close naturally or remain throughout development [55]. We envision that the proposed model could be (i) calibrated at the point of care to echocardiography data, (ii) used to simulate growth trajectories and haemodynamics with age, and (iii) used to then help clinicians assess when additional measurements are needed for cardiac monitoring. For instance, echocardiography can provide estimates of chamber size, track cardiac strain patterns and time-series estimates of valvular velocities which can all be used for subject-specific calibration [3,6,7]. Combined with data assimilation workflows [7,56], the model can then be used to help predict VSD closure and the probability of cardiovascular consequences if left untreated.

### 4.6. Limitations

There are multiple limitations for the present study. First, this initial study provides evidence that relatively simple haemodynamic models, combined with adaptation laws and VSD flow, can reproduce haemodynamics with similar magnitudes to measured data. We did not explicitly calibrate or validate our model to data obtained across VSD subjects, which is the next necessary step in transitioning our approach to a digital shadow and, eventually, a digital twin [57]. We consider a simplified model of the four heart chambers, though future modelling studies should incorporate biventricular interaction [9] to identify how septal wall motion is augmented with the defect. We have previously used sensitivity analysis on a similar compartmental model to quantify predictive uncertainty in the context of adult pulmonary hypertension [17]. However, the present model has nested levels of parameter interactions: there are parameters that affect haemodynamics at a single age and parameters that ultimately dictate how resistance, compliance and elastance change over time. Future studies that develop multiscale global sensitivity methods that explicitly account for temporal correlations are warranted. We imposed potential vascular pathologies by elevating PVR and reducing pulmonary artery compliance, though vascular remodelling should emerge because of haemodynamics. Incorporating growth and remodelling into the framework explicitly is a necessary next step [8]. We found that left-to-right shunt volume overloaded the RV, which would be corrected with a proper growth and remodelling framework. Finally, we only considered lumped-parameter representations of the systemic and pulmonary arteries and veins. Including additional compartments, such as the brain, liver or coronary circulation [7], would increase the complexity of the problem but may help identify specific relationships between VSD size and compartment-specific haemodynamics [38].

## 5. Conclusion

This study provides a computational platform for examining the effects of small-, medium- and large-sized VSDs on haemodynamics from 1 day to 2 years old. We used a relatively simplistic model with a minimal number of parameters to identify how age-dependent parametrizations, combined with various VSD sizes, alter haemodynamic loading in the cardiac chambers. Our results are consistent with prior measurements in neonates and provide an initial study for using computational modelling to understand VSD management. This is a necessary step towards the use of computer models in subject-specific management of isolated VSD and other congenital heart diseases which coincide with VSDs in neonates.

## Supplementary Material



## Data Availability

Data and models used to construct the results and figures of this manuscript can be found at the repository [58]. Supplementary material is available online [59].
