# Supplementary material for: Mathematical simulations of paediatric haemodynamics in isolated ventricular septal defect
Source: J R Soc Interface. 2026 Oct 14;23(243):20260187. doi: 10.1098/rsif.2026.0187 (PMC13682560; doi:10.1098/rsif.2026.0187)
Supplement: Supplementary file 1 [file rsif.2026.0187.zip › rsif.2026.0187/rsif20260187_si_001.docx]

**Supplementary Material to: Mathematical simulations of pediatric hemodynamics in isolated ventricular septal defect**

Mitchel J. Colebank^1,2*^, Alfonso Limon^3,4^, Anthony Chang^3,4^, Brandon Wong^4,5^, Wyman Lai^4,5^, Hamilton Baker^6^

^1^Department of Mathematics, University of South Carolina, Columbia, SC

^2^Department of Biomedical Engineering, University of South Carolina, Columbia, SC

^3^Mi4, Children’s Hospital of Orange County, Anaheim, CA

^4^Children’s Hospital of Orange County, Anaheim, CA,

^5^University of California, Irvine, Irvine

^6^Medical University of South Carolina, Charleston, SC

**Hemodynamic Predictions in a Newborn Female**

Model predictions are provided in Supplementary Figure 1 for 2-day old newborn. The same outputs from the manuscript are provided.

Figure S1. (a-c) Simulations in a two-day old newborn, including atrial and ventricular pressure-volume loops (a), vascular pressures (b), and valvular flows (c).

**Effects of Right Ventricular Stiffening**

We show how increased right ventricular stiffening reduces volume overload in VSD simulations. This highlights the potential role of right ventricular adaption to moderate and large size VSDs.

Figure S2. Effects of RV remodeling on hemodynamic predictions in the LV (a) and RV (b). The medium- and large-sized VSDs are simulated without RV adaptation, and then with a doubling of minimum elastance, $E_{min}$, in the RV to reflect stiffening of the ventricle.
